# Ultra-Deep Pyrosequencing (UDPS) Data Treatment to Study Amplicon HCV Minor Variants

**DOI:** 10.1371/journal.pone.0083361

**Published:** 2013-12-31

**Authors:** Josep Gregori, Juan I. Esteban, María Cubero, Damir Garcia-Cehic, Celia Perales, Rosario Casillas, Miguel Alvarez-Tejado, Francisco Rodríguez-Frías, Jaume Guardia, Esteban Domingo, Josep Quer

**Affiliations:** 1 Liver Unit, Internal Medicine, Lab. Malalties Hepàtiques, Vall d'Hebron Institut Recerca-Hospital Universitari Vall d'Hebron (VHIR-HUVH), Barcelona, Spain; 2 Roche Diagnostics SL, Sant Cugat del Vallès, Spain; 3 CIBER de Enfermedades Hepáticas y Digestivas (CIBERehd) del Instituto de Salud Carlos III, Madrid, Spain; 4 Universitat Autònoma de Barcelona, Bellaterra, Spain; 5 Centro de Biología Molecular Severo Ochoa (CBM), UAM, Madrid, Spain; 6 Biochemistry Unit, HUVH, Barcelona, Spain; Kliniken der Stadt Köln gGmbH, Germany

## Abstract

We have investigated the reliability and reproducibility of HCV viral quasispecies quantification by ultra-deep pyrosequencing (UDPS) methods. Our study has been divided in two parts. First of all, by UDPS sequencing of clone mixes samples we have established the global noise level of UDPS and fine tuned a data treatment workflow previously optimized for HBV sequence analysis. Secondly, we have studied the reproducibility of the methodology by comparing 5 amplicons from two patient samples on three massive sequencing platforms (FLX+, FLX and Junior) after applying the error filters developed from the clonal/control study. After noise filtering the UDPS results, the three replicates showed the same 12 polymorphic sites above 0.7%, with a mean CV of 4.86%. Two polymorphic sites below 0.6% were identified by two replicates and one replicate respectively. A total of 25, 23 and 26 haplotypes were detected by GS-Junior, GS-FLX and GS-FLX+. The observed CVs for the normalized Shannon entropy (Sn), the mutation frequency (Mf), and the nucleotidic diversity (Pi) were 1.46%, 3.96% and 3.78%. The mean absolute difference in the two patients (5 amplicons each), in the GS-FLX and GS-FLX+, were 1.46%, 3.96% and 3.78% for Sn, Mf and Pi. No false polymorphic site was observed above 0.5%.

Our results indicate that UDPS is an optimal alternative to molecular cloning for quantitative study of HCV viral quasispecies populations, both in complexity and composition. We propose an UDPS data treatment workflow for amplicons from the RNA viral quasispecies which, at a sequencing depth of at least 10,000 reads per strand, enables to obtain sequences and frequencies of consensus haplotypes above 0.5% abundance with no erroneous mutations, with high confidence, resistant mutants as minor variants at the level of 1%, with high confidence that variants are not missed, and highly confident measures of quasispecies complexity.

## Introduction

Hepatitis C virus (HCV) is a small, enveloped virus with a 9.6-kb positive single-stranded RNA genome. Like most RNA viruses, HCV evolves rapidly due to high mutation rates of 10^−3^ to 10^−4^ mutations per nucleotide per genomic replication (natural evolutionary rate of 1.5×10^−3^ base substitutions/site/year) [1], [2] through an error-prone RNA polymerase without proofreading capacity and high-level viral replication (10^12^ virions per day in an infected patient) [3], [4]. Consequently, in infected individuals, HCV replicates and circulates as a quasispecies composed of a complex mixture of different, but closely related genomes [5] that undergoes continuous changes due to competitive selection [6], [7] and cooperation [8] between arising mutants.

Genetic diversity plays a key role in the biology and medical treatment of viruses. It is well known that antiviral treatment with pegIFN+ribavirin at initiation of HCV infection achieves sustained virological response (SVR) rates of 80% to 90%, whereas treatment during the chronic phase yields rates below 50%. This is because the viral population shows low heterogeneity at the beginning of infection, whereas complexity increases during the chronic phase, thus facilitating resistance to antiviral therapy. The emergence of viral resistance to new direct antiviral agents in chronic HCV patients is associated with selection of minor subpopulations present in the HCV quasispecies. The number of amino acid substitutions needed and the genetic and phenotypic barriers involved are determinant parameters of how likely selection of a drug-resistant mutant will be. A central issue in HCV is whether drug-resistant mutants preexist in HCV populations (ie, in treatment-naive patients), and whether the presence of minority subpopulations with mutations that decrease sensitivity to direct antiviral agents (DAAs)s would help to determine the treatment regimen. Sanger sequencing and ultra-deep pyrosequencing have identified mutations that confer resistance to protease inhibitors in patients who have not been exposed to these drugs [9]. In any case, an international expert panel (HCV Drug Development Advisory Group; HCV DRAG) has implemented detailed recommendations for resistance testing during the clinical evaluation of new DAAs. pre-treatment samples should be rigorously analyzed by ultra-deep population sequencing to identify simple mutation patterns of known or novel pre-existing variants and to provide the baseline (consensus) sequence for mutations emerging at later time points [10].

Before the appearance of next-generation sequencing (NGS), the genetic diversity of a viral population and co-occurrence of particular mutations could only be assessed by cloning and Sanger sequencing. Because this is high-cost, time-consuming, labor-intensive approach, few studies have surveyed viral populations in appreciable detail, with analysis of only 20 to few hundred viruses per sample. This limitation is overcome by using massive parallel sequencing with 454 ultra-deep pyrosequencing (UDPS) [11]. This platform is the most popular choice because it enables the longest reads (about 400 bp, and sooner 900 bp). Single molecules can be sequenced from a PCR product (amplicon) with a high coverage of 10,000 or more reads. The main challenge of this technology is the development of tools that will enable confident data treatment and analysis.

In this study, we developed a data treatment pipeline based on analysis of HCV NS3 clone mixes, and assessed the reproducibility of the three 454 platforms (GS-FLX, GS-Junior, and GS-FLX+) when applied to characteristic HCV quasispecies from patient serum samples. Despite the growing number of publications with NGS data treatment methods [12]–[14] some application-oriented solutions for 454 data management are still needed. Specifically in the virology field, various solutions have been explored for error treatment and haplotype reconstruction, using NGS shotgun sequences [12], [15]–[22]. Haplotype reconstruction is a challenge because in most situations, solutions are not unique. The growing capacity to sequence by amplicons of 400 to 500 nt or even longer on the GS-FLX+ platform makes unnecessary, in most cases, the haplotype reconstruction in target sequences in order to identify whether two or more mutations are located within a single amplicon. In general, analysis by amplicons is much simpler than analysis by shotgun sequences when a viral quasispecies population has to be characterized by haplotype abundance. In this case, from the perspective of data treatment, an error treatment workflow suffices to obtain reliable results.

Different methods have been proposed to deal with PCR+ sequencing errors. One of the first is a Poisson statistical model for errors distinguishing homopolymeric and non-homopolymeric tracts [23], where errors are supposed to be fully random and independent of position and strand, followed by more complicated statistical models [15], [16], [21], [24], in which some basic assumptions are still required. When these assumptions fail and we have to account for position- and strand-specific errors, application of a model requires knowledge of the error profile of the sequence being analyzed, which is almost impossible when dealing with highly variable viruses [25], even when a substantial part of the sequencing resources are dedicated to control samples. Other ways, such as the one proposed in the present study, are ad hoc solution-oriented algorithms based on a series of filters [18], [26]–[29]. We recently reported a comparative study of UDPS and cloning in HBV samples, in which most of the data treatment pipeline described here was developed [30].

Beyond the data treatment, reliable detection of minor variants in amplicons depends on experimental issues related to the use of optimal primers to avoid nonspecific binding, PCR drift, and PCR bias, the use of high fidelity enzymes with very low cross-over incidence, and accurate purification and quantification [18].

## Materials and Methods

Two sets of samples have been used in the experimental work:

- The first set involved several genotype 1a clones of well known sequence that helped us identify and fix the limits of error frequency of the complete methodology. We used a clone from the HCV NS3 region that was named wild type (wt), and from it we generated two clones (named M1 and M3) that differed from the wt in two genomic positions of the sequence. As a simulation of a putative result observed in nature, we site-directed mutated two genomic positions related to antiviral resistance. The three clones were mixed PCR amplified and pyrosequenced.
- The second set involved 2 patients of genotype 1b from which we studied 5 amplicons of the complete NS5A region. The aim of this study, was to measure the reproducibility of the 454 methodology in real patient samples using three different 454 platforms (GS-Junior, GS-FLS and GS-FLX+) after applying the error filters developed from our clonal/control study.

### 1. Samples and Methods for Control-Clonal Studies

#### Wild type clone (wt)

Clone B2b (GenBank accession number: **EF613712**) from the NS3 HCV region obtained from liver biopsy of a chronic HCV patient was selected and considered the wild type (wt) reference sequence. The clone, which did not carry any substitutions related with antiviral resistance to any known inhibitor, was inserted into the commercial plasmid, pCR4Blunt-TOPO (Invitrogen, Carlsbad, CA, USA). Wt positive sense HCV-RNA was obtained by in vitro transcription after PstI linearization using T7 RNA polymerase (SP6/T7 Transcription Kit, Roche, Mannheim, Germany). The amino acid combination at positions 54 and 155 was T54 (ACT)-R155 (AGA).

#### Site-directed mutagenesis to produce clones with antiviral resistance mutations

Two antiviral-resistant mutants were obtained by site-directed mutagenesis using the Quick Change Lighting Site-Directed Mutagenesis commercial kit (Agilent-Stratagene, Santa Clara, CA, USA), as specified by the manufacturers. Primer pairs used to generate the mutant M1 [S54 (TCT)-R155 (AGA)] were as follows:

*Sense* (5′-C ATT AAC GGA GTG TGC TGG ***T***CT GTC TAC CAC GGG GCC GGA AC-3′)

*Antisense* (5′-GT TCC GGC CCC GTG GTA GAC AG***A*** CCA GCA CAC TCC GTT AAT G-3′). To generate the mutant M3 [T54 (ACT)-K155 (AAA)], the following primers were used:

*Sense* (5′-GGA CAC GCC GTA GGC ATT TTC A***A***A GCC GCG GTG TGC ACC CGT GG-3′)

*Antisense* (5′-CC ACG GGT GCA CAC CGC GGC T**T**T GAA AAT GCC TAC GGC GTG TCC-3′)

Positive sense HCV-RNA from clones M1 and M3 was obtained by in vitro transcription after PstI linearization using the T7 Ribomax Express Large Scale RNA Production System kit (Promega, Madison, WI, USA).

Three RNAs were ultimately obtained: the wt and the two mutants, M1 and M3.

#### Mutant mixes (QAv1.2 and QAv1.3)

RNAs from M1 and M3 were quantified by Ribogreen and mixed at different percentages (**Table S1 in File S1**). Each mix was amplified by nested RT-PCR, as specified for patient samples (see below), using specific NS3 primers adapted for the 454 UDPS platform:

NS3up3543 5′-CGTATCGCCTCCCTCGCGCCA**TCAG**ACTTTCTTAGCAACCTGCATTAA-3′ (48)

NS3d3962 5′-CTATGCGCCTTGCCAGCCCGC**TCAG**GGACCTCATGGTTGTCTCTAGG-3′ (47)

The bold face letters indicate the barcode used for signal calibration in 454 pyrosequencing.

#### RT-PCR-Nested amplification

HCV RNA was extracted from 140 µL of plasma/serum by automatic RNA extraction (TNAI) or manual RNA extraction, using the Qiagen Total RNA extraction kit (Qiagen, Hilden, Germany), as specified by the manufacturers. The measures to prevent contamination suggested by Kwok and Higuchi [31] were strictly applied.

For patient samples, amplifications were focused on the NS5A region. The complete NS5A region was amplified using 5 overlapping primer pairs (**Table S2 in File S1**). Reverse transcription was performed with AccuScript High Fidelity reverse transcriptase and PCR reactions with Pfu Ultra II High-Fidelity enzyme using Accu-Script PfuUltra II Fusion HS (Agilent-Stratagene, Santa Clara, CA, USA) according to the manufacturer's recommendations. The first PCR reaction involved standard amplification with specific primers covering the region of interest.

Briefly, 1 µL of all extracted HCV-RNA was mixed with 10× Bufferx, 1 µL dNTP, and 20 pmol of antisense PCR primer (30 pmol if degenerate primers were used) to a final volume of 8 µL. After 5 min of denaturation at 65°C, 1 µL of DTT together with 1 µL of AccuScript HF-RT were added to a final volume of 10 µL. Reverse transcription was performed at 25°C for 10 min followed by 42°C for 30 min, and maintained at 4°C until the PCR reaction (GeneAmp 2700 PCR system, Applied Biosystems, Foster City, CA, USA). One to five microliters of cDNA was mixed with 5 µL of 10× specific buffer, 1 µL of dNTP, 1 µL (20 pmol, or 30 pmol if degenerate primers were used) of each forward and reverse primer and finally, 1 µL of PfuUltra II High fidelity DNA polymerase to a final volume of 50 µL. After denaturing for 1 min at 95°C, 40 cycles of 30 seconds at 95°C, 30 seconds at 55°C, and 3 min at 68°C were performed, with a final 10-min step at 68°C.

Five microliters from the PCR were amplified by a second PCR, using specific primers and following the above-described PCR reaction conditions.

### 2. Massive sequencing analysis of NS3 and NS5A region, primers

To compensate for random sampling and substitution errors, nested RT-PCRs were performed in triplicate and then mixed. Amplification products were analyzed by 1.8% agarose gel electrophoresis and negative controls (amplifications in the absence of RNA) were included in parallel to assure absence of contamination by template nucleic acids. The final PCR yields 400- to 500-bp fragments. PCR products were purified in agarose gel using the QIAquick Gel Extraction kit (Qiagen, Valencia, CA, USA) as specified by the manufacturers, quantified using the PicoGreen assay (Invitrogen, Carlsbad, CA, USA), and analyzed for quality using the BioAnalyzer DNA 1000 LabChip (Agilent, Santa Clara, CA, USA) prior to UDPS. We used titanium chemistry, which enables sequencing of 400- to 500-nt fragments.

### 3. Experimental design

We performed two sets of experiments: a clone control to test the results of our pipeline and set a noise level, and a real-life amplicon sample to assess UDPS reproducibility using the three available platforms.

The first set of experiments aimed at finding patterns of PCR+UDPS errors in order to develop filters that would be useful for HCV amplicon data sequencing analysis. The experiment included 16 samples in a plate divided into 16 lanes. Four mixes of two clones with two nucleotide differences near the two edges and in different proportions (with the minority at 5, 1, 0.5 and 0.1%) were replicated four times (see above, and **Table S1 in File S1**). The design intended to obtain an average of 10,000 reads per each strand (forward and reverse) and sample, deemed sufficient to detect minor variants below 1%.

A second experiment, at a higher sequencing depth, aimed at evaluating the reproducibility of the UDPS data, and compared the findings on HCV samples, sequenced on three different instruments (GS-Junior, GS-FLX, and GS-FLX+), with GS-FLX+ being located in a different laboratory with different technicians. The experiment consisted in sequencing five NS5A amplicons from two patients. The plate was divided into two regions with one patient in each region. On FLX Junior, only one amplicon from one patient was sequenced to obtain a comparable sequencing depth. Amplification of each sample was done by three independent PCRs that were later mixed. The products sequenced on each platform were produced by the same PCR mix; hence, there was no variability before the sequencing protocol.

### 4. Filtering UDPS reads

We followed the data treatment described in (13) with minor refinements (F**igure 1**). Briefly, the reads in each fasta file obtained from 454 were demultiplexed to obtain a fasta file with the reads corresponding to each sample (combination of a Multiplex IDentifier -MID-, primer, and strand). These reads were subsequently submitted to a data quality filter with allowances to correct up to 2 Ns and 3 gaps. The accepted and eventually repaired reads were collapsed into haplotypes with the corresponding frequencies. Haplotypes observed on the fw and rv strands were intersected, excluding uncommon sequences, and the frequencies were added. The final step consisted in removing the fw+rv consensus haplotypes with an abundance below the problem-specific background noise level, with the aim of limiting as much as possible haplotypes carrying PCR and sequencing errors. We found that for HCV amplicons 400 to 500 nt in length with a sequencing depth of not less than 10, 000 reads per strand, a level of 0.5% assures with high confidence that no erroneous haplotypes will pass the final filter (except for sporadically occurring chimeras).

**Figure 1:**
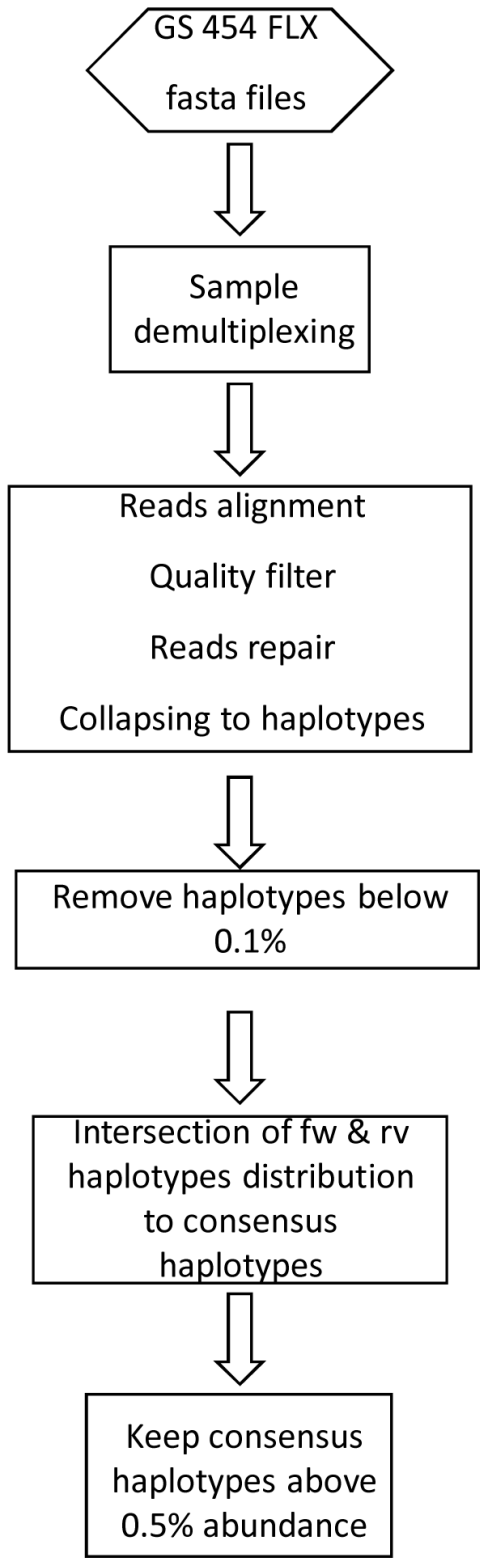
UDPS data treatment workflow to obtain error-free HCV haplotypes as a sampling of the actual HCV viral quasispecies.

### 5. Substitution errors

Analysis of the combined RT+PCR+UDPS substitution errors was done on the haplotypes after the quality filter, accepting haplotypes with an abundance above 0.01% and a minimum of two reads. We distinguished both strands and homopolymeric and non-homopolymeric tracts.

### 6. Characterization of HCV quasispecies complexity

Complexity of the viral quasispecies was quantified by Shannon entropy(Sn), which is a measure of haplotype diversity regardless of the number of mutations implied, mutation frequency (Mf), which measures the diversity with respect to the most highly represented sequence (master haplotype), and nucleotide diversity(Pi), which takes into account the average number of mutations between each pair of haplotypes n the viral population. Each of these parameters explains a different part of the mutation space occupied by a quasispecies, and all are relevant when considering mutation barriers to resistance.

Montserrat plots [30], density plots of the frequencies of mutants respect to the dominant haplotype, were used to visualize the population diversity in comparison with the dominant haplotype in terms of the number of mutations.

### 7. PCR primer bias

The putative bias in sequencing introduced by the use of suboptimal primers in the PCR stage can be brought to light in multiple amplicon studies. When amplicons overlap each other, we can check how the previous and next amplicon see the forward and reverse primers of each amplicon, respectively (**Figure S1**). An absence of mutations in the primer regions would indicate that there is no bias, and the presence of mutations would imply preferential amplification of some variants over others, and therefore, bias in estimating the distribution of the viral population.

### 8. Probability of missing variants

Once a reliable abundance threshold above the noise level for true variants is established, the probability that variants will not be missed in the sampling process will depend on the coverage or sequencing level. Binomial distribution was used to estimate the confidence at which we were able to detect variants at a given abundance (above the threshold) as a function of the sequencing depth.

### 9. Statistics and computation environment

The percent deviation between replicates was computed as the difference between replicate abundance for each haplotype relative to the corresponding mean abundance. Likewise, the relative errors for the measures of quasispecies complexity were computed as the difference between replicates with respect to the mean value. In comparisons with three replicates, however, deviations were characterized by CV values.

All data processing was done on the open source R environment [32], using Bioconductor [24] and the Biostrings library [33] for pattern matching and sequence alignment, and the R functions we developed for this purpose. The R scripts are available upon request.

## Results

### 1. PCR and UDPS errors

**Figure 2:**
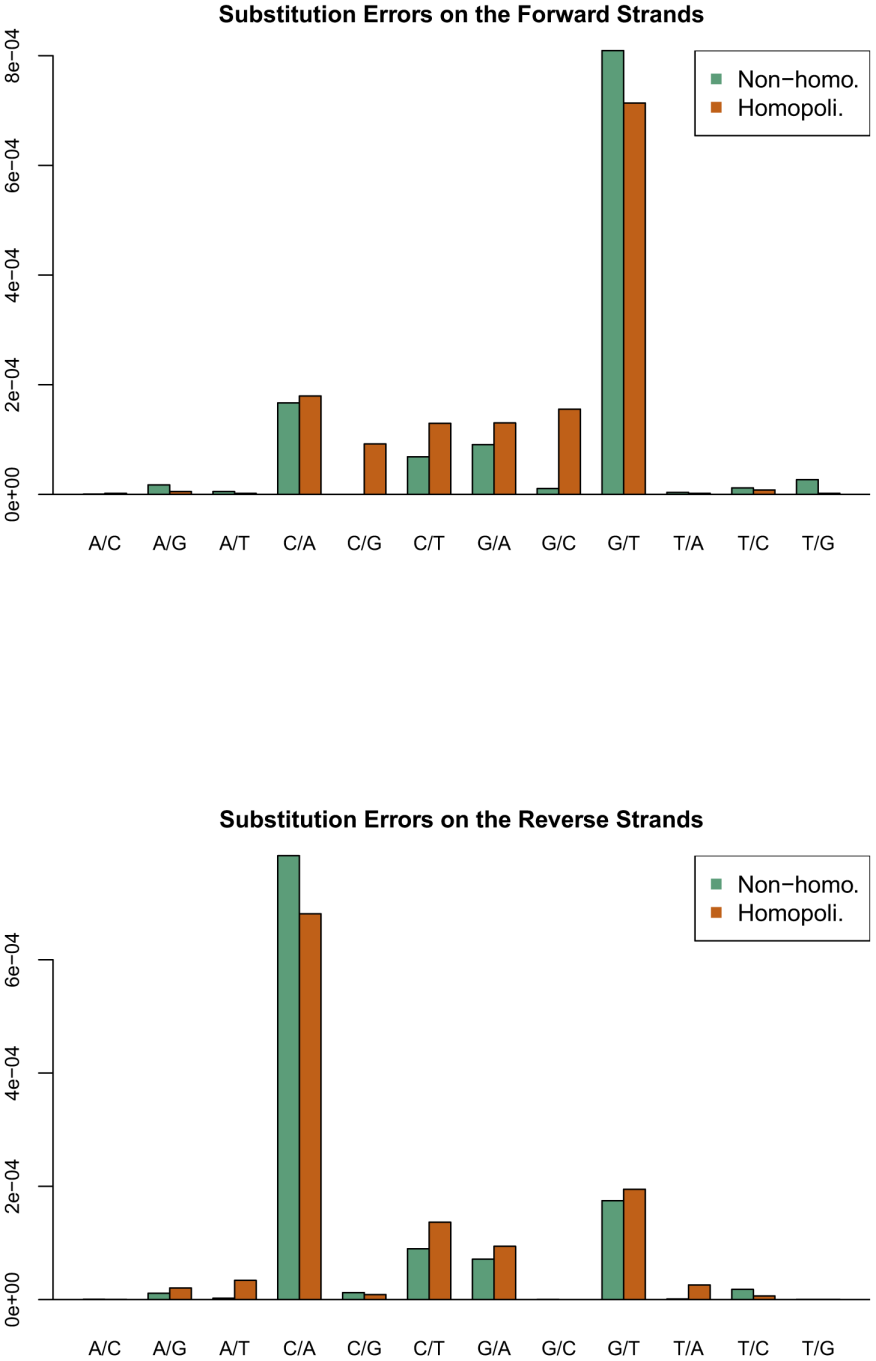
Observed substitution errors in the experiment with clones. a) Forward strand b) Reverse strand. The dominant G to T on fw (C to A on rv) are tied to in vitro transcription of the (+) chain in the plasmid, which is used as HCV viral source.

The first experiment was performed with a simple HCV quasispecies formed by two clones with one nucleotide difference near each amplicon end, in different percentages, to determine the substitution error rates profile. We obtained a substitution error rate matrix for each strand (fw and rv), and also differentiated between/homopolymeric and non-homopolymeric regions. A site was designated as homopolymeric when it was within a four-nucleotide or greater homopolymer or at its borders; that is, flanking the homopolymer. We found that the substitution error rates of the fw strands were different from those of the rv strands (**Figure 2**), for both homopolymeric and non-homopolymeric regions. As HCV has a single-stranded RNA genome, the differences observed are easily explained by the relatively high error rate of in-vitro retrotranscription to double-strand cDNA, but they also could imply that the error rates in each site were not only dependent on the nucleotide and homopolymeric status, but also on the surroundings. The most common error in the forward strands was a substitution of G to T at a rate of 776.90 per million G sequenced, followed by a substitution of C to A at a rate of 171.85 per million C sequenced, whereas in the reverse strands, the rates of the same substitutions were 182.56 and 748.87, respectively. Hence, when the reverse and complemented strands were added to the forward strands, the G to T substitution error was, by far, the most prominent.

The most relevant errors observed were in homopolymeric regions and caused gaps instead of substitutions. This was mainly seen in homopolymers of 5 nucleotides or more, but also in some of only 4 nucleotides. Of note, the gaps observed in a particular site were seen in the fw or rv strand, but not in the complementary strand. A gap in both strands was only observed in large homopolymers, of at least 7 nucleotides. Therefore, alignment of the reads found against a reference sequence is fundamental to enable correction for selective gaps and to obtain high overlapping between what is observed on the forward and reverse strands (ie, high-confidence haplotypes).

As substitution errors appear to be independent of the surroundings, the best tool we have at hand to control for errors is comparison of what is on the forward strands against what is on the reverse strands. **Table 1a** shows the number of correct and erroneous haplotypes and polymorphic sites found set on analysis by point mutations common to fw and rv (analysis by columns on the alignment) with a mutation abundance cut-off of 0.25%, and an analysis by consensus haplotypes (analysis by rows on the alignment) with a haplotype abundance cut-off of 0.25%. **Table 1b** shows the results when the cut-off was increased to 0.5%. The numerous erroneous polymorphic sites and haplotypes observed at the 0.25% abundance cut-off completely disappeared at 0.5%, and the analysis by columns seemed to be slightly more sensitive to minor point mutations.

**Table 1:** PCR and UDPS errors.

Table 1a. 0.25%	By columns F (.01)+Av+F(.25)			By rows F (.10)+I+F(.25)		
	Point Mutations			Point Mutations		
	Err	OK	Haplotypes	Err	OK	Haplotypes
Lane 1	2	2	-	2	2	5
Lane 5	5	2	-	6	2	9
Lane 9	0	2	-	0	2	3
Lane 13	7	2	-	7	2	10
Lane 2	12	0	-	12	0	13
Lane 6	18	0	-	20	0	21
Lane 10	2	0	-	0	0	1
Lane 14	4	0	-	5	0	6
Lane 3	10	0	-	13	0	14
Lane 7	2	0	-	3	0	4
Lane 11	13	0	-	13	0	14
Lane 15	6	0	-	6	0	7
Lane 4	11	0	-	10	0	11
Lane 8	5	0	-	6	0	7
Lane 12	12	0	-	15	0	16
Lane 16	4	0	-	7	0	8

Table 1b. 0.5%	By columns F (.01)+Av+F(.50)			By rows F (.10)+I+F(.50)		
	Point Mutations			Point Mutations		
	Err	OK	Haplotypes	Err	OK	Haplotypes
Lane 1	0	1	-	0	1	2
Lane 5	0	2	-	0	1	2
Lane 9	0	2	-	0	2	3
Lane 13	0	2	-	0	2	3
Lane 2	0	0	-	0	0	1
Lane 6	0	0	-	0	0	1
Lane 10	0	0	-	0	0	1
Lane 14	0	0	-	0	0	1
Lane 3	0	0	-	0	0	1
Lane 7	0	0	-	0	0	1
Lane 11	0	0	-	0	0	1
Lane 15	0	0	-	0	0	1
Lane 4	0	0	-	0	0	1
Lane 8	0	0	-	0	0	1
Lane 12	0	0	-	0	0	1
Lane 16	0	0	-	0	0	1

Experiment with clones. Point mutations and haplotypes found when cutting consensus point mutations (analysis by columns) or consensus haplotypes (analysis by rows) at 0.25% abundance (**Table 1a**) at a cut-off of 0.50% abundance (**Table 1b**). Err indicates the number of false segregating sites, and OK the number of true segregating sites.

The estimated abundances of the true minor variants in haplotypes above 0.01% and a minimum of 2 reads did not differ from those obtained in the analysis by rows or columns at a cut-off of 0.5%. On the other hand, when analyzing the consensus haplotypes obtained from the analysis by rows at a 0.5% cut-off, when no errors survive, we observe the sequence of the M3 mutant as dominant and the chimeras M1∶M3 and M3∶M1 as minor variants, but no sequence corresponding to the M1 mutant is found. See **Table 2** with the number of reads by sample and strand after each data treatment step, and the observed distribution of haplotypes. In fact, the M1 haplotype was not observed at any abundance cut-off.

**Table 2:** Number of reads per sample and strand at each data treatment step, and haplotype distribution at a consensus haplotype abundance >0.5%.

							Observed haplotypes				Nominal
	Identified	QF plus Correction	0.1% Filter	Intersection		0.5% Filter	M3	M1∶M3	M3∶M1	Others	M1
1 fw	12582	11376	10880	10744	28895	27602	99,22	0,78			5
1 rv	20853	19235	18214	18151							
2 fw	13514	12301	11598	11392	29964	27217	100				1
2 rv	21869	19975	18805	18572							
3 fw	16494	15047	14177	13988	26063	23941	100				0,5
3 rv	14184	12919	12251	12075							
4 fw	13017	11740	11088	10925	29272	26905	100				0,1
4 rv	21533	19826	18649	18347							
5 fw	12450	11315	10746	10518	27109	25604	99,26	0,74			5
5 rv	19250	17677	16786	16591							
6 fw	11130	9767	9222	9163	21046	18234	100				1
6 rv	14269	12804	12069	11883							
7 fw	6332	5613	5420	5332	30574	29527	100				0,5
7 rv	29004	27089	25556	25242							
8 fw	9084	8045	7791	7637	23609	22504	100				0,1
8 rv	18723	17168	16213	15972							
9 fw	15022	13965	12918	12765	25262	24517	98,46	0,95	0,58		5
9 rv	14594	13443	12608	12497							
10 fw	12490	11480	10683	10381	24153	24012	100				1
10 rv	16240	14900	13880	13772							
11 fw	14141	12825	12066	11811	25581	22875	100				0,5
11 rv	16413	14840	13901	13770							
12 fw	13189	11822	11132	10951	24780	22194	100				0,1
12 rv	16467	14955	14013	13829							
13 fw	7554	6516	6170	6066	20055	18714	98,06	1,02	0,92		5
13 rv	17048	15468	14490	13989							
14 fw	12429	11368	10750	10559	27509	25723	100				1
14 rv	19862	18232	17172	16950							
15 fw	4894	4127	3973	3825	22323	20819	100				0,5
15 rv	22561	20431	18884	18498							
16 fw	8198	7209	6793	6501	17714	16647	100				0,1
16 rv	13472	12130	11341	11213							
**Reads**	478862	435608	410239	403909		377035					
**Relative Yield**		91,0%	94,2%	98,5%		93,3%					
**Global Yield**		91,0%	85,7%	84,3%		78,7%					

To detect minor variants with a high confidence level at an abundance below 1%, it is important to reach a minimum sequencing depth of 10,000 reads in the forward and reverse HCV strands. Our results with clones showed that lower coverages (under 10,000 reads) generated doubtful results and, for instance some minor variants were detected in the forward but not in reverse strands (data not shown).

### 2. UDPS reproducibility

#### 2.1 A single amplicon in three replicates

A single amplicon of the NS3-protease region was sequenced on the GS-Junior and GS-FLX and GS-FLX+ platforms. The sequencing depth in this experiment was a mean of 38,800 reads per strand (min 24,700, max 47,600). The number of reads for the forward and reverse strand in each sample was approximately balanced, except for the sample sequenced in the 454 FLX, which incidentally showed a number of reads of the forward strand of only 52% of that obtained for the reverse strand. The number of reads after each data treatment step is shown in **Table 2**. The low-frequency filter embedded in the quality control step, eliminating all haplotypes with just one read, caused the greatest impact, taking around one-third of the reads. The second largest impact was caused by the haplotype filter below 0.1% abundance. Both filters were responsible for eliminating around 50% of the reads in the fasta file obtained from the 454 sequencer. After filtering by an abundance above 0.1%, the reads belonging to haplotypes common to the forward and reverse strands were greater than 97% for GS-FLX and GS-FLX+, and 95% for GS-Junior.

The measures of quasispecies complexity resulting from each platform, when considering the consensus haplotypes (CH) filtered above 0.25% and 0.5% are shown in **Table 3a and 3b**. The CV was below 5% for mutation frequency and nucleotide diversity, and below 2% for Shannon entropy, at both abundance cut-offs. The high imbalance in the number of reads obtained for the forward and reverse strands in the sample sequenced by GS-FLX caused the largest deviation.

**Table 3:** Reproducibility: Quasispecies complexity measures for a single amplicon replicated on three platforms, located in two laboratories.

3a. FCH 0.25%	Hpl	Eta	S	Mf×10^3^	Sn	Pi×10^3^
JR UCTS	46	22	21	2,4977	0,6239	4,249
FLX UCTS	43	23	22	2,3547	0,6147	4,021
FLX+CRAG	47	21	20	2,5805	0,6339	4,360
CV				4,61%	1,54%	4,11%

3b. FCH 0.50%						
JR UCTS	25	14	14	2,1500	0,6351	3,666
FLX UCTS	23	13	13	2,0445	0,6315	3,493
FLX+CRAG	26	15	15	2,2118	0,6492	3,765
CV				3,96%	1,46%	3,78%

Hpl stand for number of haplotypes, Eta for number of mutations, S for number of polymorphic sites, Mf for mutation frequency, Sn for normalized Shannon entropy and Pi for nucleotide diversity.

The population overlap between platforms when CH were filtered above 0.25% and 0.5% are depicted in **Tables S3** and **S4 in File S1**, respectively. Mutant abundance at each polymorphic site along with the CV are shown in **Table 4** **.** Mean CV was 4.86% (0%–13.58%) for CH at 0.5%, and 9.53% (1.77%–40.0%) for CH at 0.25%.

**Table 4:** Reproducibility: Comparison of mutant abundance at each polymorphic site for a single amplicon replicated on three platforms, located in two laboratories when considering all CH>0.5%.

pos	UCTS Jr	UCTS FLX	CRAG FLX+	Mean	SD	CV
7223	25,89	25,41	25,82	25,71	0,2593	1,01%
7598	14,61	13,63	14,99	14,41	0,7017	4,87%
7484	11,95	11,06	12,22	11,74	0,6070	5,17%
7346	11,47	11,37	11,66	11,50	0,1476	1,28%
7532	10,30	9,15	10,56	10,00	0,7504	7,50%
7303	3,62	3,71	3,78	3,70	0,0802	2,17%
7229	2,08	1,95	2,09	2,04	0,0781	3,83%
7370	1,24	1,14	1,27	1,22	0,0713	5,86%
7455	1,24	1,14	1,27	1,22	0,0681	5,59%
7442	1,19	0,92	1,16	1,09	0,1480	13,58%
7459	0,73	0,74	0,83	0,77	0,0551	7,18%
7579	0,73	0,74	0,83	0,77	0,0551	7,18%
7340	0,59	0,62	0,60	0,60	0,0169	2,82%
7475	0,58		0,58	0,58		
7246			0,59	0,59		
					Mean	4,86%
					Min	0%
					Max	13,58%

#### 2.2 Ten amplicons on the GS-FLX and GS-FLX+

The NS3 region of two patients, covered by five slightly overlapped amplicons was sequenced on 454-FLX and GS-FLX+. Both samples came from a common PCR, which was a mix of products from three independent PCRs. The high variability of this region produced a rich population of mutants, with abundant haplotypes at distances as far as 11 nucleotides within the same amplicon.

The number of reads after each data treatment step in the two replicates are shown in **Tables S5** and **S6 in File S1**. The low frequency filter embedded in the quality control step, eliminating all haplotypes with just one read, caused a loss of a 15% to 20% of the demultiplexed reads. Roughly, 70% of the demultiplexed reads survived the 0.1% abundance filter, of which 98% belonged to haplotypes in common to the two strands. Consensus haplotypes with an abundance above 0.5% amounted to roughly 60% of the demultiplexed reads.

The measures of quasispecies complexity resulting from the two platforms, when considering CH filtered above 0.5% are presented in **Table 5**. The mean relative difference was around 5% for mutation frequency and nucleotide diversity, and 2% for Shannon entropy.

**Table 5:** Reproducibility: Measures of quasispecies complexity for ten amplicons replicated on GS-FLX and GS-FLX+ located in two different laboratories.

FLX @ UCTS		Hpl	Eta	S	Mf·10^3^	Sn	Pi·10^3^
Sample 1	Ampl 1	31	25	25	2,1118	0,5807	4,019
	Ampl 2	24	22	22	1,1382	0,5069	2,062
	Ampl 3	22	16	15	1,9863	0,5467	3,626
	Ampl 4	24	16	15	1,9620	0,5130	3,539
	Ampl 5	23	13	13	2,0445	0,6315	3,493
Sample 2	Ampl 1	25	20	20	2,9966	0,6095	4,064
	Ampl 2	20	19	19	0,7650	0,4703	1,492
	Ampl 3	16	14	14	2,1070	0,4955	2,772
	Ampl 4	16	15	15	0,7122	0,3541	1,375
	Ampl 5	20	13	12	2,0502	0,7163	3,263

FLX+ @ CRAG							
Sample 1	Ampl 1	31	25	25	2,2400	0,5959	4,248
	Ampl 2	27	24	24	1,2418	0,5216	2,262
	Ampl 3	24	15	14	2,3277	0,5616	4,240
	Ampl 4	24	17	16	1,8692	0,5080	3,390
	Ampl 5	26	15	15	2,2118	0,6492	3,765
Sample 2	Ampl 1	23	19	19	2,9992	0,6153	4,041
	Ampl 2	21	20	20	0,8050	0,4866	1,570
	Ampl 3	15	13	13	2,0973	0,4975	2,745
	Ampl 4	16	15	15	0,7210	0,3554	1,393
	Ampl 5	23	14	13	2,0883	0,7040	3,311

Rel. Diffs.					Mf%		Pi%
Sample 1	Ampl 1	0,0%	0,0%	0,0%	5,9%	2,6%	5,5%
	Ampl 2	11,8%	8,7%	8,7%	8,7%	2,9%	9,2%
	Ampl 3	8,7%	6,5%	6,9%	15,8%	2,7%	15,6%
	Ampl 4	0,0%	6,1%	6,5%	4,8%	1,0%	4,3%
	Ampl 5	12,2%	14,3%	14.3%	7,9%	2,8%	7,5%
Sample 2	Ampl 1	8,3%	5,1%	5,1%	0,1%	1,0%	0,6%
	Ampl 2	4,9%	5,1%	5,1%	5,1%	3,4%	5,1%
	Ampl 3	6,5%	7,4%	7,4%	0,5%	0,4%	1,0%
	Ampl 4	0,0%	0,0%	0,0%	1,2%	0,3%	1,3%
	Ampl 5	14,0%	7,4%	8,0%	1,8%	1,7%	1,5%
Mean					5,2%	1,9%	5,2%
SD					4,6%	1,1%	4,5%

Data from CH>0.5%.

Hpl haplotypes, Eta number of mutations, S number of polymorphic sites, Mf mutation frequency, Sn normalized Shannon entropy, Pi nucleotide diversity.

**Figure 3:**
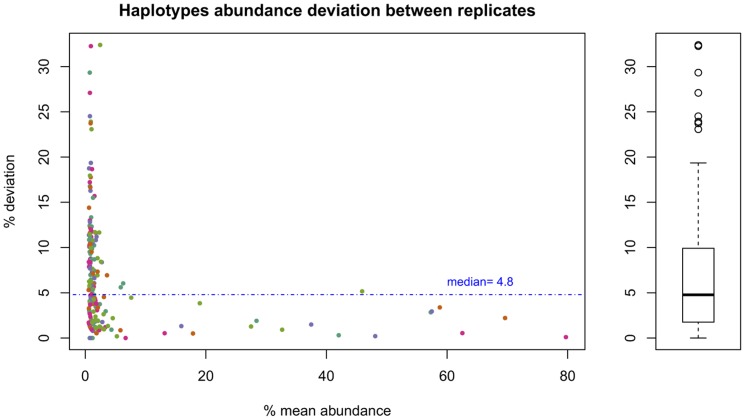
Reproducibility: Haplotype abundance relative difference vs haplotype mean abundance, and box plot of relative differences. Ten samples in FLX and FLX+. Dot color identifies the amplicon to which the haplotype belongs.

The population overlap between platforms for a given sample ranged from 96% to 100%, with a mean of 99%, and values were slightly higher using the 0.5% cut-off than the 0.25% one (**Tables S7** and **S8 in File S1**). The relative deviations in abundance for all CH above 0.5% common to the two platforms showed a median of 4.8%. Deviations increased exponentially at abundances below 2% (**Figure 3**).

### 3. PCR bias by suboptimal primers

The primers were designed based on multiple alignment of consensus sequences, usually obtained by Sanger method, which are available at public access databases, such as GenBank or Los Alamos. They may be pangenotypic, in which case some degenerations may be introduced to cover the variations in some positions, or they may be subtype-specific with or without degeneracy. HCV is an RNA virus with a high replication error rate, which introduces complexity in the quasispecies composition. The quasispecies concept in itself limits the usefulness of Sanger and consensus sequences in the design of primers. Despite this limitation, no better information is currently available, and there is no alternative to the risk of introducing PCR amplification bias due to the use of suboptimal primers.

We checked the adequacy of our primers for the five amplicons from NS5A by inspecting how they are seen on amplicon overlapping regions (**Figure S1**). Although subtype-specific primers were used, there was substantial discrepancy in mutation abundances in the overlap of amplicons 1 and 2 for patient 1 (**Figure S2**). Exactly the same discrepancy and at the same level was observed in GS FLX at UCTS and GS-FLX+ at the CRAG laboratory. Almost no discrepancies were observed in the other amplicon overlaps. Mutations were seen in most of the primer regions, and in as many as 4 polymorphic sites on the reverse primer for amplicon 2 (data not shown). Some of the primers showed no polymorphic sites, but there were some differences (as many as 3) with respect to the designed primer. In patient 1, only one primer, amplicon 3 forward, showed no polymorphic sites or differences relative to the designed primer.

### 4. Probability of missing variants

By establishing the abundance threshold above noise level for a coverage of 10,000 fw and rv reads at 0.5%, all haplotypes with less than 50 reads after filtering and correction would be excluded. The probability of missing a variant at a true 1% in the population is given by the binomial distribution as the probability of obtaining 0 to 49 reads in a sample of 10,000, when the probability of sampling such reads from the viral population is 0.01. Based on these numbers, the probability of missing a variant at 1% in the viral population at this sequencing coverage is 1.03×10^−8^, and the probability of missing a real variant at an abundance of 0.5% is 0.48. When the quality and noise filter reduces coverage to 60% of all reads, the probability of missing a real variant based on raw coverage of 10,000 reads is 6.33×10^−6^ at 1% abundance and 0.48 at 0.5%.

Hence, when achieving a coverage of 10,000 fw and rv reads, the data treatment pipeline assures with high confidence that no false polymorphic site is retained, and that the probability of missing a real variant at 1% abundance is extremely low.

## Discussion

### 1. Error rates and data treatment

Because of its inherent variability, sequencing quasispecies samples to estimate the composition of an RNA viral population is an enormous challenge and requires the use of specific tools. The greatest contribution of a platform able to sequence long amplicons resides in the possibility to estimate the distribution of haplotypes in a viral population, and not just point mutation abundance. Knowledge of this distribution enables study of putative associations between mutations within the same amplicon, and better tracing of the emergence of a resistant mutant, thanks to the contribution of compensatory mutations, which could balance the impaired fitness of single mutants.

Despite the availability of platforms that can sequence amplicons longer than 400 bases, the errors introduced during the process of retrotranscription to DNA, the required PCR steps, and the sequencing process itself, make it difficult to distinguish what is real from what is artefactual. We used a series of basic clone controls to establish a probabilistic model that would help in recognizing errors. We found that the assumption of independence of site in the error profile did not hold, what means that a realistic statistical model for error substitutions rates produced by PCR+UDPS based on empirical data and taking into account surroundings and dependencies, would require sequencing samples containing all possible combinations of nucleotides more than once, which is impractical. Instead, we placed our attention on a system of filters tuned to exclude almost all false polymorphic sites when studying mutations present at abundances below 1%. The filters are based on the assumption of background noise plus the existence of higher order errors that are dependent on the surroundings and that can easily be detected when sequencing the forward and reverse strands. These higher order errors may be caused by secondary structures that persist at the working temperatures or by other interactions with the surrounding nucleotides.

The filters showed that when a 0.5% cut off was used for consensus haplotype abundance, no false polymorphic sites were identified, whereas at a cut-off of 0.25%, a number of false-positives emerged. In addition, it was necessary to reach a consistent level of 10,000 reads in both the forward and reverse strands to reach reliable results at abundances below 1%. As was mentioned above, a critical step is confrontation of the forward and reverse sequences to easily identify erroneous haplotypes and polymorphic sites.

The method relies heavily on having a reference sequence that provides a reliable pattern for alignment, which can be used to correct the gaps alignment may produce on the dominant haplotype. Gaps on haplotypes below the dominant one can be then corrected, if required, by the contents of the dominant haplotype, itself. Correction of gaps and Ns must be restricted as much as possible, especially when studying highly variable regions of the viral quasispecies, at the risk of introducing false mutations. Nevertheless, the complexity of such regions requires correction of some gaps at the risk of losing most of the sequenced reads.

The use of a high fidelity enzyme (pfu) to minimize the PCR errors seems to be related to the impossibility to detect the minor variant M1, but instead their recombinant chimeras with the M3 mutant, that is M1∶M3 and M3∶M1. The highly imbalanced abundance between the two mutants in the mixes causes progressive dilution of the minor variant as recombination progresses along the PCR cycles until it vanishes completely. To limit the production of PCR recombinants, a more highly processive enzyme can be used at the risk of higher error rates, as fidelity and processiveness are always in balance. We are currently optimizing the PCR process with the aim of restricting recombinants while maintaining high fidelity.

Each of the four sample mixes at different percentages was prepared independently in four replicates, from a single quantification of the two original clones. Although the four replicates showed high reproducibility, we were far from the nominal percentage values. We suggest that low accuracy of DNA quantification and the difficulty of preparing quite balanced mixes of DNA samples are among the main causes of this difference.

Despite the deviations introduced by recombination, point mutation abundances for the true polymorphic sites remained the same on fw and rv haplotypes filtered at 0.01%, on consensus point mutations filtered at 0.5%, and on consensus haplotypes filtered at 0.5% (see **Table S9 in File S1**).

The filters presented are simple and easy to implement, and can be tuned using a small number of parameters, so they can be quickly adapted to case-specific experiments. With a sequencing depth of both strands of at least 10,000 reads, and an abundance cut-off of 0.5%, no false polymorphic sites are identified.

### 2. Reproducibility

Once a data treatment workflow that enabled identification of point mutations and haplotypes at 1% abundance with high confidence had been established, we judged it important to assess the reproducibility of UDPS results when sequencing the same serum sample on different platforms and in different laboratories. Reproducibility is a major concern when dealing with any of the so-called *omics* technologies. The non-negligible cost involved restricted our study to replicas of one amplicon on 454 FLX, 454 FLX+, and 454 Junior, and to a replica of ten samples on 454 FLX and 454 FLX+. The Junior and FLX are located at the UCTS facilities in our center, whereas the GS-FLX+ is located at the CRAG laboratory in the Autonomous University of Barcelona.

Beyond characterization of point mutations, the ability to obtain high-confidence haplotypes enables computation of measures of quasispecies complexity that are related to the severity and degree of chronicity of an infection. Therefore these measures can be considered prognostic factors. The 5% CV observed for mutation frequency and nucleotide diversity, and the 2% for Shannon entropy showed high reproducibility for obtaining complexity measures. The only alternative to UDPS for this purpose is traditional cloning, which, at a comparable cost, falls far short of this accuracy and reproducibility. The measures are highly sensitive to the number and frequency of haplotypes identified and the very high overlap of populations estimated from the two platforms (mean 99%, minimum 96% in the 10 samples) guarantees good accuracy.

To the three platforms, the mean CV of 4.9% for common point mutations (maximum 13.6%), also showed a very high degree of reproducibility. The least reproducible point mutations were those very near the 0.5% cut-off, a fact related to the sampling process.

When considering the 10 samples replicated in GS-FLX and GS-FLX+, located in different laboratories, we observed a mean relative difference of common CH abundance of 4.8% comparable to the CVs observed for the point mutations. The relative differences increased suddenly for abundances below 2%

### 3. PCR bias due to suboptimal primers

The high variability of the HCV genome makes it quite difficult, if not impossible, to design primers that can guarantee freedom from bias in the PCR amplification process beforehand. By definition, all primers designed by current methods will provide suboptimal primers for HCV, except for the very highly conserved regions. This is a real limitation that cannot be overcome with current knowledge based on consensus sequences. Use of overlapped amplicons is needed to check the degree of bias afterwards. In some circumstances, the only way to guarantee a sufficiently robust approach to determine the true viral population composition would be to redesign the primers based on a previous sequencing experiment with the same sample.

### 4. Clinical applicability

Assessing the pre-existance of resistance mutations at baseline and monitoring resistance during treatment (non-response during treatment, at breakthrough or relapse) is becoming a key factor in deciding the most suitable HCV antiviral combination therapy [10], [34], [35]. In case of treatment failure, it will help to decide the new treatment. Since antiviral-resistant mutants are often less replication-competent than the corresponding wild-type (wt) viruses, they represent minority mutants in the absence of drug therapy. However, the concomitant presence of compensatory (or secondary) mutations in the same sequence may allow a more efficient replication. Currently, there is not any alternative routine methodology able to detect and quantify HCV resistant variants (neither for HBV or HIV) other than ultra-deep sequencing, specially due to the high variability generated by HCV. We have performed this work with subtypes 1a and 1b, but UDPS technology can be easily adapted to other subtypes. We have successfully designed and tested primers for other subtypes on the NS3 and NS5A regions, using the same data treatment pipeline.

Here we have shown that any of the three platforms of Ultra-deep pyrosequencing is a highly reproducible for detection of minority mutants, provided that the amplicons are long enough and with high sequencing depth. By using control samples, the pipeline has proved to be highly efficient at detecting minority variants below 1%. The probability to miss a minority variant at 1% given a sequencing depth not lower than 10,000 reads fw and 10,000 rv is 1.03×10^−8^.

## Conclusions

By using clones and serum samples from HCV patients, we developed a UDPS data treatment workflow for amplicons from the RNA viral quasispecies which, at a sequencing depth of at least 10,000 reads per strand, enables the following to be obtained:

✓ sequences and frequencies of consensus haplotypes above 0.5% abundance with no erroneous mutations, with high confidence,
✓ resistant mutants as minor variants at the level of 1%, with high confidence that variants are not missed,.
✓ measures of quasispecies complexity with an estimated CV below 5%, and
✓ an expected population overlap between replicates above 95%.

The pipeline also provides a means for checking for bias introduced by inadequate primers in multiamplicon studies.

The algorithm is based on a series of filters and repairs that preserve 50% to 70% of the demultiplexed reads, and whose parameters allow different levels of stringency to accommodate case-specific problems.

## Supporting Information

Figure S1 Coloured scaled strips indicate each one of the five overlapping amplicons used to amplify the complete NS5A region of HCV. Vertical names refer to specific primers. Numbers along the continuous coloured strip indicate the number of reads studied from each amplicon after applying the algorithm.
(PDF)

Figure S2 Abundance of mutations in the overlapping regions of each amplicon compared with the next one(s). Abscissa (x) indicates nucleotide mutated position in NS5A and ordinate the number of substitutions. Pink column indicates the number of substitutions at the 3′ overlapping end of the amplicon and blue column at the 5′ overlapping end.
(PDF)

File S1 Supplementary Tables are combined into a single excel file named File S1. Each Table is labeled as Table S1 to Table S9 and separated in individual excel sheets. **Table S1** - Proportion of the minority mutant in each mix, and number of molecules from which the emPCR started. Experiment QAv1.2 contains two technical replicates of each of four mixes, both starting from 10^4^ and 10^5^ molecules. Experiment Av1.3 consists of four independent replicates of the same four mixes. **Table S2** - Primers used for the retrotranscription, and to amplify five overlapping amplicons of the NS5A region. The 5th amplicon has primers specific for subtype 1a and 1b. **Table S3** - Consensus haplotypes filtered at 0.25% for three replicates of one NS5A patient amplicon on the three 454 platforms. A) Number of reads. B) Overlapping population between replicates. C) Common haplotypes. D) Common polymorphic sites. **Table S4** - Consensus haplotypes filtered at 0.5% for three replicates of one NS5A patient amplicon on the three 454 platforms. A) Number of reads. B) Overlapping population between replicates. C) Common haplotypes. D) Common polymorphic sites. **Table S5** - Number of reads after each data treatment step on each of the 10 NS5A amplicons sequenced on the 454 FLX. **Table S6** - Number of reads after each data treatment step on each of the 10 NS5A amplicons sequenced on the 454 FLX+. **Table S7** - Overlapping populations between replicates of patient samples per amplicon, sequenced with the 454 FLX and FLX+, considering the consensus haplotypes filtered at 0.5%. **Table S8** - Overlapping populations between replicates of patient samples per amplicon, sequenced with the 454 FLX and FLX+, considering the consensus haplotypes filtered at 0.25%. **Table S9** - Observed reads and relative abundances of true mutants. A) Haplotypes filtered at 0.1%, followed by forward and reverse haplotype intersection, followed with a further abundance filter at 0.5%. B) Haplotypes filtered at 0.1%, followed by forward and reverse point mutation intersection, followed with a further abundance filter at 0.5%. C) On forward and reverse strands, after filtering haplotypes at 0.1% abundance.
(XLSX)
