# Supplementary figures and images for: Ultra-Deep Pyrosequencing (UDPS) Data Treatment to Study Amplicon HCV Minor Variants
Source: PLoS One. 2013 Dec 31;8(12):e83361. doi: 10.1371/journal.pone.0083361 (PMC3877031; doi:10.1371/journal.pone.0083361)

amplicons overlap: FLX-15 (CH 0.50%)

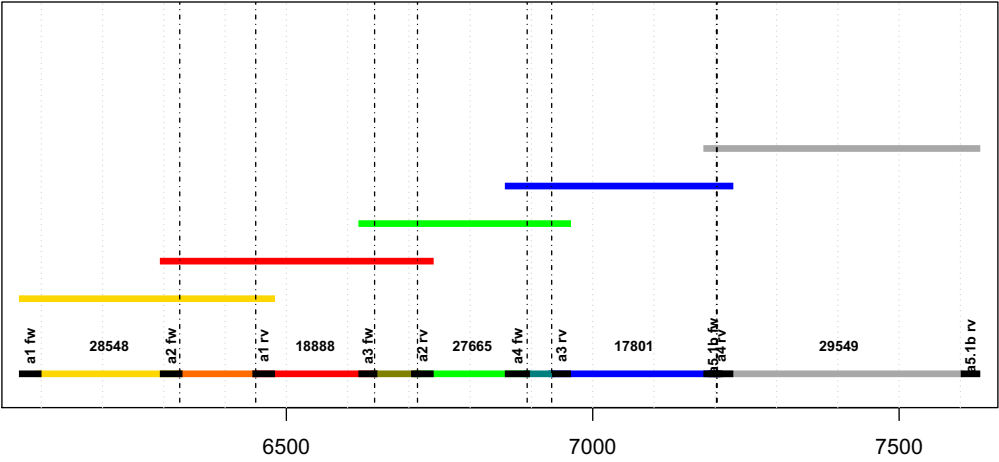

Supplement: Figure S1 — Coloured scaled strips indicate each one of the five overlapping amplicons used to amplify the complete NS5A region of HCV. Vertical names refer to specific primers. Numbers along the continuous coloured strip indicate the number of reads studied from each amplicon after applying the algorithm. (PDF) [file pone.0083361.s001.pdf]

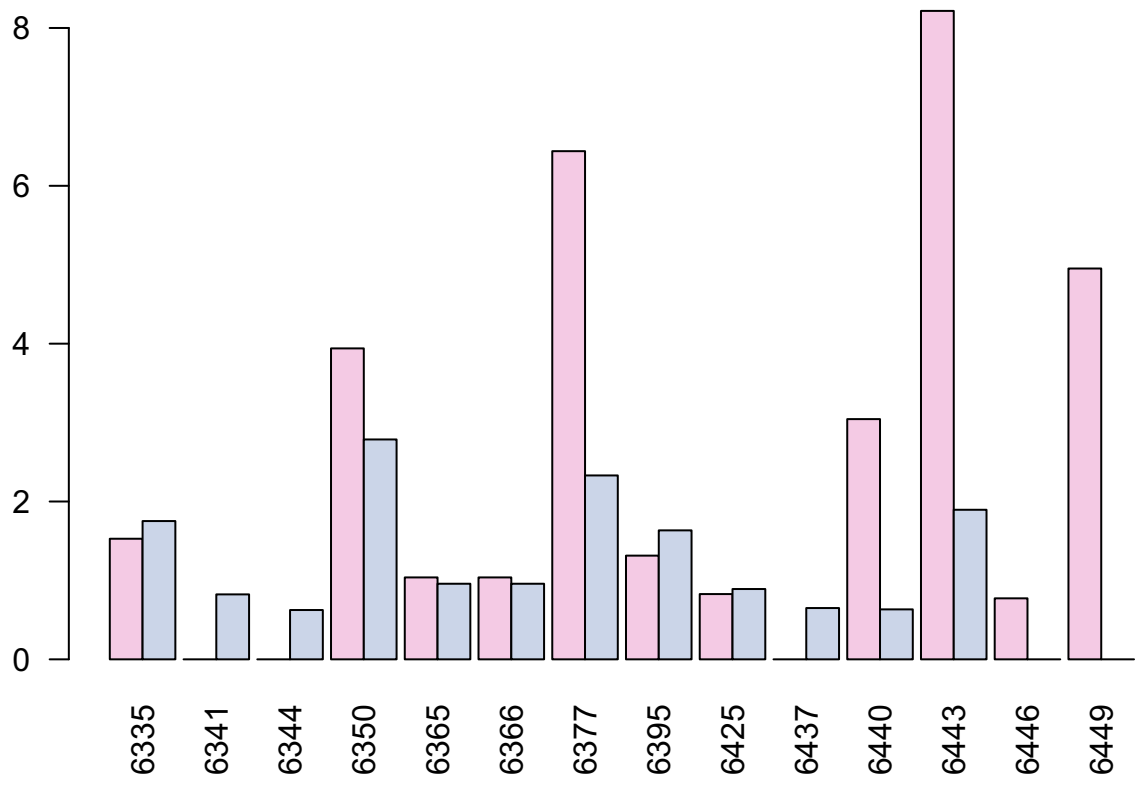

Supplement: Figure S2 — Abundance of mutations in the overlapping regions of each amplicon compared with the next one(s). Abscissa (x) indicates nucleotide mutated position in NS5A and ordinate the number of substitutions. Pink column indicates the number of substitutions at the 3′ overlapping end of the amplicon and blue column at the 5′ overlapping end. (PDF) [file pone.0083361.s002.pdf]
